# A GCU-SAM Enhanced Transformer for Fault Diagnosis of Rotating Machinery

**DOI:** 10.3390/s26154771

**Published:** 2026-07-27

**Authors:** Jing Li, Lei Hu, Peng Luo

**Affiliations:** 1 School of Transportation and Electrical Engineering, Hunan University of Technology, Zhuzhou 412007, China; 2 School of Engineering Science, Shandong Xiehe University, Jinan 250107, China; 3 Hunan Provincial Key Laboratory of Health Maintenance for Mechanical Equipment, Hunan University of Science and Technology, Xiangtan 411201, China

**Keywords:** rotating machinery, fault diagnosis, deep learning, gated convolution, self-attention mechanism

## Abstract

Fault signals in rotating machinery typically manifest as long time-series data embedded with local high-frequency impulses. Traditional deep learning methods often struggle to simultaneously capture these transient local impacts and model long-term global degradation features. To address this challenge, this paper proposes a novel GCU-SAM enhanced Transformer for intelligent fault diagnosis. The proposed network integrates a Gated Convolutional Unit (GCU) with a Self-Attention Mechanism (SAM). By introducing the GCU as a local inductive bias prior to the global attention module, the model dynamically captures and purifies local impulse responses via reset and update gates. Subsequently, a cascaded multi-head self-attention mechanism models long-sequence global evolution trends, forming an integrated framework for local fine-grained perception and global correlation modeling. Validated on the CWRU bearing and SEU gearbox datasets, the proposed architecture achieves superior diagnostic performance with an average F1-score of 99.01%. Compared to representative baselines, including 1D-CNN, BiLSTM, and the standard Transformer, the GCU-SAM significantly boosts diagnostic accuracy and effectively overcomes the early-stage optimization oscillations inherent in pure attention mechanisms. By achieving a deep multi-scale fusion of local abrupt changes and global degradation trends, the model exhibits exceptional feature-clustering discriminative capability and convergence stability, providing a robust and highly accurate solution for the intelligent fault diagnosis of complex rotating machinery.

## 1. Introduction

Rotating machinery serves as the core transmission equipment in modern industrial applications, and its operational reliability directly impacts production safety and efficiency. In recent years, data-driven methods based on deep learning have achieved remarkable progress in the field of intelligent fault diagnosis owing to their end-to-end adaptive feature mining capabilities, gradually replacing traditional signal processing methods that heavily rely on expert experience [1,2,3,4].

However, when applied to core equipment characterized by strong nonlinearity and multi-component signal coupling (such as complex planetary gearboxes) [5], existing deep learning architectures exhibit prominent structural limitations in balancing multi-scale feature extraction. Specifically, restricted by a fixed local receptive field, one-dimensional convolutional neural networks (1D-CNN) struggle to directly capture long-range temporal dependencies [6]. Conversely, self-attention architectures that possess excellent global modeling capabilities (such as the Transformer) lack a local inductive bias [7], resulting in diminished recognition sensitivity to local signal continuity and weak transient changes. This limitation not only frequently leads to the loss of high-frequency transient impact features associated with early weak faults during long-range mapping but also subjects pure attention networks to severe optimization difficulties and convergence oscillations during the early stages of training [8]. Consequently, simultaneously balancing local high-frequency impact feature extraction and long-sequence global evolution modeling under complex operational environments remains a critical challenge in this field [9].

Although several CNN–Transformer hybrid architectures have been proposed for fault diagnosis, they predominantly rely on rigid, cascaded convolutional layers that treat all features with equal importance. These conventional methods often struggle to effectively separate transient fault impulses from background noise, particularly under complex operational conditions. To address this, our work introduces the GCU-SAM enhanced transformer. The innovation of our approach is twofold: first, we introduce the Gated Convolutional Unit (GCU) as a physically informed local prior that acts as a learnable band-pass filter, which distinguishes our model from existing static feature-fusion architectures. Second, by utilizing reset and update gates, the GCU performs Dynamic Signal Conditioning—selectively amplifying transient fault-related signatures while suppressing non-stationary noise. This ‘soft-selection’ capability ensures that the global self-attention mechanism operates on ‘purified’ features where fault impulses are highlighted, providing a robust and methodologically rigorous diagnostic framework that significantly outperforms existing cascaded models.

Furthermore, ensuring the reliability and interpretability of these advanced deep learning architectures under variable operating conditions has become a critical research frontier. Recent studies have emphasized the necessity of interpretable frameworks for the fault diagnosis of rotor systems. For instance, Rezazadeh et al. [10] proposed a fine-tuning deep learning framework that effectively palliates data distribution shift effects in rotary machines, employing novel visualization techniques for internal activation patterns to elucidate the sequential feature learning process. Such interpretable approaches highlight the importance of not only achieving high diagnostic accuracy but also understanding internal feature representations. Inspired by these advancements, our proposed GCU-SAM architecture explicitly isolates the signal-adaptive preprocessing (via the GCU gating mechanism) from the global dependency modeling. This architectural transparency ensures that the self-attention mechanism operates on highly discriminative, task-relevant fault signatures, thereby naturally enhancing the overall interpretability of the diagnostic process.

### Contributions

- Construction of a dual-path temporal feature learning framework that integrates “local fine-grained perception and global association modeling,” effectively compensating for the standard Transformer’s shortcomings in local feature extraction for non-stationary vibration signals.
- Introduction of a lightweight local feature extraction module based on a gating mechanism—the Gated Convolutional Unit (GCU). By using reset and update gates to adaptively regulate information transfer within the local receptive field, it precisely captures and enhances high-frequency local impact responses and fine-grained degradation features in time-series signals.
- Combination of positional encoding with a multi-head self-attention mechanism to deeply mine and decouple global long-range dependencies of the enhanced local feature sequence. While preserving long-term evolutionary pattern modeling, this framework significantly improves the model’s sensitivity to local abnormal impact signals, offering a novel perspective and a feasible technical solution for high-precision fault diagnosis of rotating machinery under practical engineering applications.

## 2. Related Work

Data-driven intelligent diagnosis has emerged as the dominant paradigm for contemporary equipment health monitoring [10,11,12,13,14]. Among various network topologies, 1D-CNN, by virtue of their local receptive fields and weight-sharing mechanisms, are widely used to extract high-frequency local impact features from vibration signals [15,16,17,18]. However, convolution-based architectures struggle to capture global long-range temporal dependencies across extended time intervals.

To overcome the bottleneck of long-distance temporal modeling, the Transformer architecture, based on the self-attention mechanism, has been increasingly adopted within the fault diagnosis domain [19,20]. Nevertheless, several recent studies [21,22,23] indicate that because the Scaled Dot-Product Attention (SDPA) mechanism treats all time steps within a sequence equally, it lacks a local inductive bias. Consequently, when processing non-stationary high-frequency vibration signals, it tends to attenuate the local transient responses that carry early weak fault information, and is highly susceptible to optimization oscillations during the early stages of training.

To facilitate multi-scale feature extraction, lightweight hybrid architectures that integrate the local perception advantages of CNNs with the global modeling capabilities of Transformers have emerged as a prominent research focus [24,25]. For instance, CNN–Transformer hybrid networks incorporating wavelet decomposition or empirical mode decomposition have successfully enhanced the fused representation of multi-scale signals. Furthermore, the introduction of dynamic gating mechanisms has proven effective in filtering out redundant background noise and isolating core degradation features [26].

However, existing hybrid architectures predominantly rely on cascaded standard convolutional layers. When processing complex amplitude- and frequency-modulated components, these cascaded structures are prone to incorporating redundant noise and lack an adaptive mechanism to regulate information flow. Motivated by these considerations, the GCU-SAM enhanced Transformer proposed in this paper incorporates a gated convolutional unit equipped with a “soft selection” regulation capability. While preserving the temporal topological structure, it dynamically weights crucial local features, thereby providing a purer and more discriminative representational foundation for long-range self-attention modeling.

Furthermore, the paradigm of intelligent fault diagnosis is rapidly evolving with the emergence of Large Language Models (LLMs). Recent research has demonstrated that leveraging LLMs in industrial settings offers new possibilities for handling complex, non-stationary signals. For instance, studies such as ‘An adaptive industrial large language model for mechanical fault diagnosis under variable operating conditions’ [27] highlight that LLMs can provide robust generalization capabilities when dealing with data distribution shifts across different operating conditions. Additionally, representative works like ‘Adaptive fault diagnosis of railway vehicle on-board controller with large language models’ [28] have further validated the potential of large-scale industrial models in interpreting complex system faults. Unlike traditional deep learning models that rely solely on numerical feature extraction, these LLM-based approaches integrate multi-modal knowledge to enhance diagnostic reasoning. While our proposed GCU-SAM enhanced transformer focuses on structural optimization for high-precision local-global feature fusion, the integration of LLM-based knowledge priors represents a promising avenue for future research to further improve diagnosis under extreme variable-load conditions.

In summary, while current hybrid models typically use additive or cascaded structures for multi-scale feature extraction, they often lack a mechanism to regulate input quality, leading to ‘information masking’ under heavy noise. Our GCU-SAM architecture innovatively shifts the paradigm from static fusion to dynamic, gate-regulated feature conditioning. By explicitly isolating signal-adaptive preprocessing (via GCU) from global dependency modeling (via SAM), we ensure the model operates on task-relevant fault signatures, a distinct innovation that enhances both the robustness and the interpretability of our diagnostic framework compared to conventional hybrid networks.

## 3. Problem Formulation

Intelligent fault diagnosis of rotating machinery is a time-series multi-class pattern recognition task. Industrial equipment operates under dynamic conditions. Sensors capture continuous vibration signals. These actual physical signals often contain complex amplitude- and frequency-modulated components and background noise.

Let the one-dimensional continuous vibration time-series dataset be $D=\{x_{i},y_{i}{\}}_{i=1}^{N}$. Where $x_{i}=\left[x_{i,1},x_{i,2},\ldots ,x_{i,L}\right]\in R^{L\times M}$ represents the input sequence, consisting of variables of length, which reflects the actual physical operating status of the equipment within a specific time frame. Furthermore, $y_{i}\in \{1,2,\ldots ,K\}$ denotes the ground-truth health condition or fault category label for each sample, where K is the total number of categories. The core of the diagnostic task is to construct a nonlinear mapping model $g\left(\cdot \right)$ with high representational capacity to achieve accurate predictions of the state probability distribution in an end-to-end manner:

(1) $$\hat{y}=g(x;\theta )$$

where $\hat{y}$ denotes the posterior probability distribution of various states output by the network; θ collectively refers to the set of learnable parameters that are continuously updated iteratively during the joint optimization process of the network topology. The ultimate goal of the model training is to find the optimal parameter set $\theta^{*}$. The model achieves this by minimizing the empirical risk over the training dataset. The optimization objective function is formulated as:

(2) $$\theta^{*}=\arg\min_{\theta }\frac{1}{N}\sum_{i=1}^{N}L({\hat{y}}_{i},y_{i})$$

where $L\left(\cdot \right)$ denotes the selected loss function. This function quantifies the discrepancy between the predicted probability distribution ${\hat{y}}_{i}$ and the true label $y_{i}$. During the training phase, an optimization algorithm iteratively updates the parameter set θ. This process drives the network to accurately learn the complex mapping rules from the vibration signals.

## 4. Methods

### 4.1. General Architecture of the Proposed Model

The architecture follows a dual-stage ‘local-prioritized’ pipeline: the Signal Conditioning Stage (GCU) and the Correlation Modeling Stage (SAM). The GCU layer performs signal denoising and local impulse enhancement, effectively transforming the raw waveform into a feature-rich sequence. By explicitly isolating local feature extraction, we relieve the subsequent SAM module from the burden of learning local patterns from scratch, allowing it to focus on modeling the global degradation evolution and inter-component correlations with high-fidelity inputs. Within the core feature extraction pipeline, the proposed model introduces a local-context-prioritized modeling mechanism. First, the raw time series is fed into the local feature learning layer. Here, a lightweight Gated Convolutional Unit (GCU) efficiently extracts local features. Through its gating mechanism, the GCU adaptively regulates information flow within the local receptive field. This process precisely captures key features such as short-term dependencies, fine-grained variations, and local waveform patterns in the temporal data. At the same time, it preserves the sequential continuity and local structural integrity of the time series. Next, these enhanced local features are fed into the multi-head attention layer. This layer captures long-range global dependencies across the sequence. Consequently, it achieves a deep fusion of local features and global correlations. Finally, a Feed-Forward Network (FFN) performs non-linear mapping, dimensional transformation, and high-order abstraction on the fused features. This process further enriches the feature representational capacity. It also significantly improves the model’s fitting and diagnostic performance for complex temporal dynamics.

**Figure 1:**
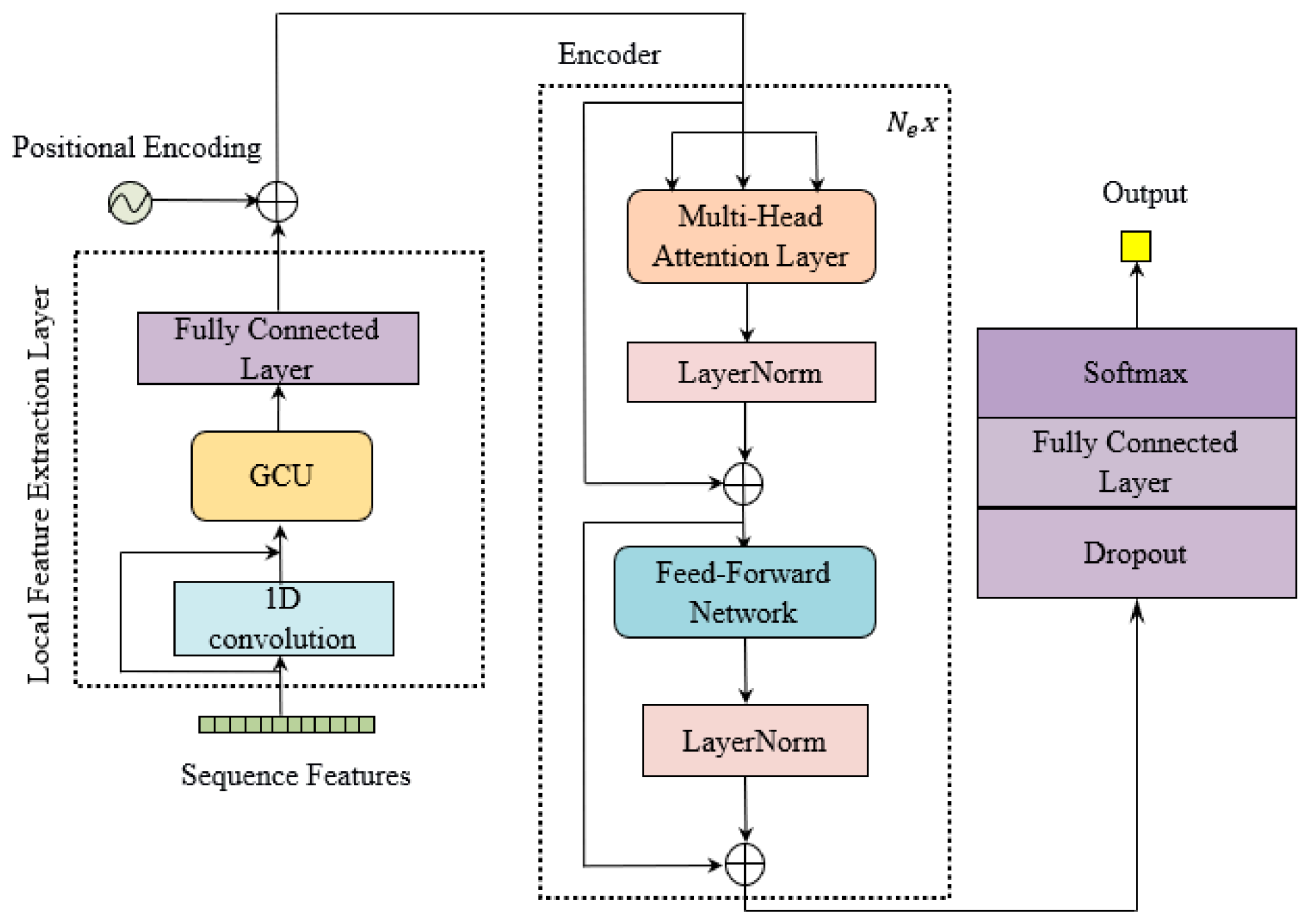
Schematic diagram of the GCU-SAM enhanced transformer network architecture.

As illustrated in Figure 1, the core feature extractor of the GCU-SAM enhanced Transformer has a clear architecture. It primarily consists of three key modules: the local feature learning layer, the multi-head attention layer, and the feed-forward neural network layer. These three modules work together in sequence to model the time-series data efficiently. This collaborative process bridges local fine-grained features with global association features.

### 4.2. Gated Convolutional Unit (GCU) for Local Feature Extraction

To compensate for the inherent deficiencies of the original Transformer in perceiving the local continuity and fine-grained impact features of time-series data, this paper introduces a Gated Convolutional Unit (GCU), inspired by the gating mechanism of LSTM, prior to the global attention mechanism.

**Figure 2:**
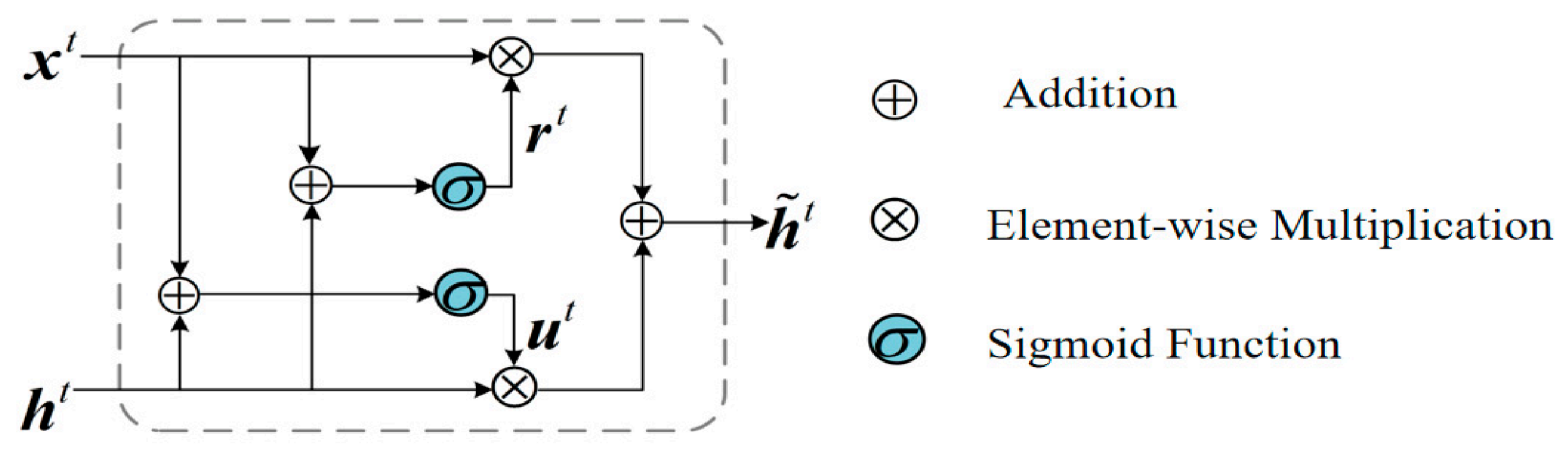
Schematic diagram of the computational workflow of the GCU.

The specific computational flow of the GCU is illustrated in Figure 2, designed to adaptively filter local features before global attention modeling. Let the input sequence be defined a $X\in R^{L\times d_{in}}$, where L is the sequence length and $d_{in}$ is the input feature dimension. First, a one-dimensional convolution captures local contextual patterns, producing an intermediate representation h:

(3) $$h=\mathrm{Conv}1D(X)\in {\mathbb{R}}^{L\times d_{out}}$$

To dynamically regulate the information flow and suppress non-stationary noise, we introduce a Reset Gate r and an Update Gate u, drawing inspiration from GRU-like gating mechanisms but adapted for local spatial features. The operations at time step t (or position t) are formulated as

(4) $$r^{t}=\sigma (W_{r}x^{t}+U_{r}h^{t}+b_{r})$$

(5) $$u^{t}=\sigma (W_{u}x^{t}+U_{u}h^{t}+b_{u})\;$$

where $\sigma \left(\cdot \right)$ denotes the Sigmoid activation function, ensuring the gate values strictly remain within the (0, 1) range, $W_{r}$, $U_{r}$, $W_{u}$, $U_{u}$ are learnable weight matrices, and $b_{r}$, $b_{u}$ are bias vectors.

Finally, the reset gate $r^{t}$ and update gate $u^{t}$ are employed to dynamically weight the original input $x^{t}$ and the intermediate convolutional representation $h^{t}$, respectively. The weighted features are then fused via element-wise addition to explicitly generate the candidate state $\tilde{h^{t}}$:

(6) $${\tilde{h}}^{t}=(r^{t}⊙x^{t})+(u^{t}⊙h^{t})\;$$

where ⊙ explicitly denotes the Hadamard product (element-wise multiplication). Through this mathematically defined gating pathway, the GCU acts as a learnable dynamic filter. The reset gate modulates the contribution of the current spatial input, while the update gate adaptively highlights the local convolutional context. This complete forward computation yields a purified candidate tensor $\tilde{H}\in R^{L\times d_{out}}$ that serves as an optimized local prior for the subsequent self-attention mechanism.

From a signal processing perspective, this gating mechanism functions as a dynamic attention-gated filter. The reset gate selectively forgets information that does not conform to the impulsive fault pattern, while the update gate adaptively highlights segments containing critical fault energy. This mechanism is physically consistent with the requirement for early-stage fault detection: it ignores stationary interference and dynamically ‘zooms in’ on the transient impacts.

### 4.3. Global Temporal Modeling via Multi-Head Self-Attention

After extracting the local context representation $\tilde{h^{t}}$ containing high-frequency impact responses via the Gated Convolutional Unit (GCU), Positional Encoding and the Multi-Head Self-Attention Mechanism are introduced into the model to further capture the global evolutionary dependencies across time steps, given that the vanilla Transformer architecture lacks the perception of sequence positional priors.

This paper first injects fixed sine/cosine absolute positional encodings into the local feature stream to reconstruct and preserve the temporal topological structure of the vibration signals. Subsequently, the features fused with positional information are fed into the multi-head self-attention layer.

**Figure 3:**
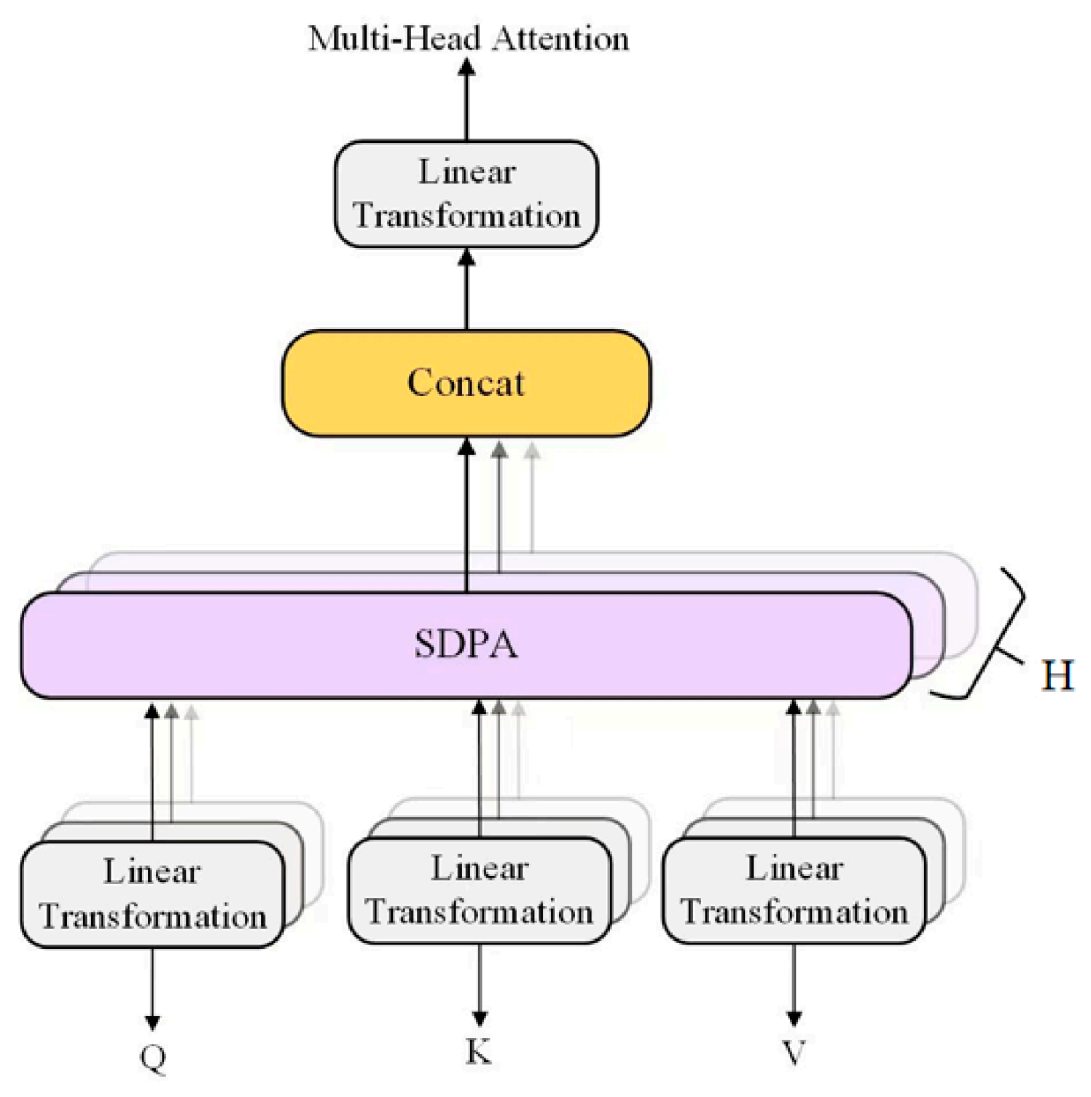
Multi-head self-attention mechanism.

Through H sets of mutually independent weight matrices, the input features are projected in parallel into distinct low-dimensional representation subspaces, generating the Query matrix Q  Key matrix K, and Value matrix V  respectively. The mathematical expression of the scaled dot-product self-attention mechanism is as follows:

(7) $$\mathrm{Attention}(Q,K,V)=\mathrm{softmax}\left(\frac{QK^{T}}{\sqrt{d_{k}}}\right)V$$

where $\sqrt{d_{k}}$ serves as the scaling factor, utilized to maintain numerical stability during deep network training. To enhance the model’s representational capacity for different feature subspaces, as illustrated in Figure 3, we employ H attention heads in parallel. The outputs of all attention heads are subsequently concatenated and passed through a linear transformation to derive the final representation:

(8) $$\mathrm{MultiAttention}(Q,K,V)=\mathrm{Concat}({\mathrm{head}}_{1},{\mathrm{head}}_{2},\ldots ,{\mathrm{head}}_{H})W^{A}$$

Compared to the standard single self-attention mechanism, the core advantage of the multi-head mechanism is its ability to decouple and extract heterogeneous degradation dependencies in parallel within multiple mutually independent subspaces. Through this multi-view concatenation mechanism, the model can simultaneously account for local high-frequency correlations, long-term degradation trends, and transient mutation patterns. This approach effectively avoids the information masking problem inherent in a single feature space and significantly improves the capture diversity of multi-scale features for non-stationary time-series signals.

### 4.4. Position-Wise Feed-Forward Network and Model Optimization

The output of the multi-head self-attention layer enters a Feed-Forward Network (FFN). This network performs higher-order nonlinear feature mapping. The FFN consists of two fully connected layers. The hidden layer uses a ReLU activation function. This enhances the model’s nonlinear representational capability.

**Figure 4:**
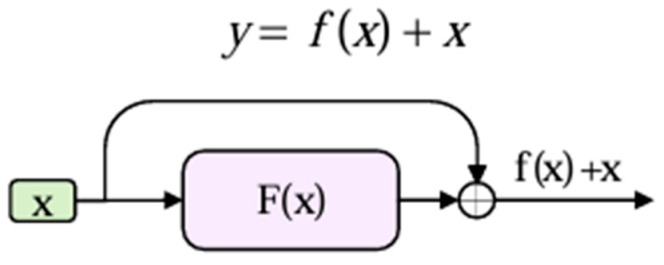
Schematic diagram of residual connection.

Deep networks often face the risk of gradient vanishing during backpropagation. To reduce this risk and speed up convergence, the model adds residual connections (as illustrated in Figure 4). These appear after both the multi-head attention mechanism and the feed-forward network. This operation adds the module’s input and its output element by element. This addition solves the gradient problem. It also helps the model preserve the original input features during parameter learning.

Finally, Layer Normalization is applied after the residual connections. This step normalizes the activation distribution within each individual sample.

The model stacks identical encoder modules. This design hierarchically extracts multi-scale global dependency features from the vibration sequences.

The GCU-SAM-enhanced Transformer encoder extracts high-dimensional abstract features. Complex networks often face the risk of overfitting. To reduce this risk, the model adds a Dropout mechanism. The features pass through this mechanism first. Next, they enter a fully connected classifier. Finally, a Softmax activation function maps them into predicted posterior probabilities for various health states. During training, the model uses Cross-Entropy as the loss function. This guides the supervised joint optimization. Its mathematical definition is as follows:

(9) $$L_{CE}=-\frac{1}{N}\sum_{i=1}^{N}\sum_{c=1}^{K}y_{i,j}\log({\hat{y}}_{i,j})$$

where  N  denotes the total number of samples involved in the computation; K  represents the total number of health state categories; $y_{i,j}$ is the ground-truth label of the sample corresponding to category (utilizing One-hot encoding); and ${\hat{y}}_{i,j}$ is the predicted probability for the corresponding category output by the network.

During parameter updating via backpropagation, this paper selects the Adam optimization algorithm. This algorithm integrates the advantages of momentum gradient descent and RMSProp, enabling the adaptive adjustment of the learning rate for each parameter. Consequently, this avoids drastic parameter oscillations and drives the network to converge to a robust local optimum efficiently and smoothly.

## 5. Experimental Validation and Analysis

### 5.1. Experimental Setup and Data Preprocessing

To comprehensively validate the diagnostic efficacy of the proposed GCU-SAM enhanced transformer architecture, this study selects two internationally recognized authoritative benchmark datasets to conduct multi-scenario experiments:

#### 5.1.1. CWRU Bearing Dataset

The dataset was collected using an accelerometer installed at the motor’s Drive End (DE), with the sampling frequency set to 12 kHz. Single-point faults were artificially introduced into the bearings using electro-discharge machining (EDM) technology, encompassing four fundamental health states: Normal (NO), Inner Race (IR) fault, Outer Race (OR) fault, and Ball (B) fault. For each fault type, three distinct damage severities—0.007, 0.014, and 0.021 inches in diameter—were subdivided. Consequently, 10 independent health/fault categories were constructed for fine-grained classification (as shown in Table 1). Furthermore, to validate the model’s stability across varying operational environments, four operating condition profiles (D1–D4), comprising different loads and rotational speeds, were selected for independent testing.

#### 5.1.2. SEU Gearbox Dataset

This dataset comes from a Drivetrain Dynamics Simulator (DDS) test bench. It records the vibration signals of a parallel shaft gearbox. The sampling frequency is 5120 Hz. Gear vibration signals have strong gear meshing frequencies and harmonic interferences. Therefore, they show higher nonlinearity than bearing signals. The dataset includes five health states: Normal (NO), Missing Tooth (MT), Root Crack (RC), Surface Wear (SW), and Chipped Tooth (CT) (as detailed in Table 2). The study evaluates the model’s robustness across different independent operational profiles. It uses two different operating conditions for the experiments: Scenario I (20 Hz rotational speed, 0 V load) and Scenario II (30 Hz rotational speed, 2 V load).

#### 5.1.3. Data Preprocessing and Dataset Partitioning

Although the CWRU and SEU datasets come from different mechanical components and have different sampling frequencies, their underlying data structures are one-dimensional continuous vibration time series. These series contain periodic impulses and nonlinear environmental noise. To meet the format requirements of deep neural networks for fixed-length input tensors and to accelerate gradient convergence, this paper adopts a unified mathematical framework for data preprocessing. Crucially, to strictly prevent data leakage, the raw datasets are first divided into a training set, a validation set, and a testing set at a ratio of 7:1:2. Only after this mutually exclusive partitioning are the overlapping sliding window technique and Z-score normalization applied to each subset independently.

1. Sliding window segmentation

**Figure 5:**
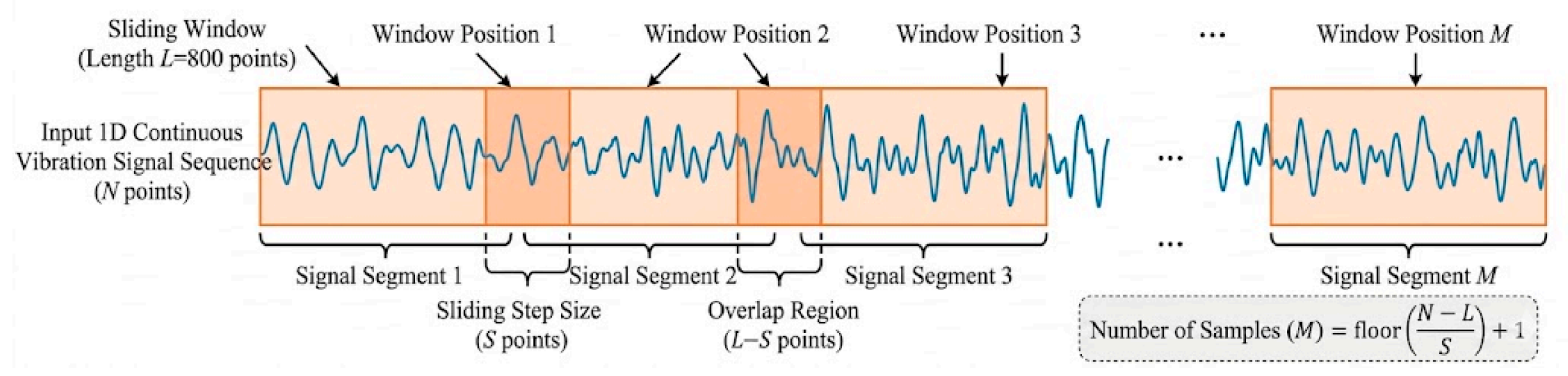
Schematic diagram of the overlapping sliding window segmentation process.

Chronological data partitioning and sliding window segmentation. To strictly prevent data leakage and ensure that highly correlated neighboring signal segments do not appear in both the training and test sets, a chronological partitioning strategy was enforced before sample generation. For each original continuous vibration recording, the raw 1D signal was split chronologically along the time axis into three mutually exclusive continuous segments. The exact lengths of these continuous segments were precisely calculated so that, after sliding window generation, the final number of generated samples would maintain a strict 7:1:2 ratio for the training, validation, and testing sets, respectively. Only after this mutually exclusive temporal separation was the overlapping sliding window technique applied independently to each continuous subset. Let the raw continuous subset length be N. The sliding window length is L, and the sliding step (stride) is S. The generated sample sequence is formally defined as:

(10) $$S_{i}=\left\{x_{i-s},x_{i-s+1},\ldots ,x_{i-s+L-1}\right\}$$

where the index i ∈{0,1,2,…,M−1} is the maximum number of samples generated under the given length and stride. Adjacent samples overlap by L−S  data points, corresponding to a 50% overlap rate, as explicitly illustrated in Figure 5. After the chronologically partitioned splitting, we obtained the exact final sample counts reported. For the CWRU dataset (L=800, S=400), this protocol yielded exactly 70 training samples, 10 validation samples, and 20 testing samples per class, totaling 100 samples per class. For the SEU dataset (L=1024, S=512), it yielded exactly 350 training samples, 50 validation samples, and 100 testing samples per class, totaling 500 samples per class. This strict sequence confirms that no overlapping windows exist across the subsets.

2. Z-score Normalization

Under variable operating conditions, step changes in motor load and speed cause severe fluctuations in vibration amplitudes. To eliminate the influence of dimensional differences and baseline shifts, this study applies Instance Z-score normalization to each dynamically segmented sample independently. The mathematical expression is

(11) $$x_{norm}^{i}=\frac{x^{i}-\mu^{i}}{\sigma^{i}}$$

where $\mu^{i}$ and $\sigma^{i}$ represent the local temporal mean and standard deviation computed exclusively within the current single sample sequence $x^{i}$. This sample-specific normalization approach stabilizes the input distribution without requiring global dataset statistics, providing unambiguously standardized input for the GCU-SAM enhanced transformer.

### 5.2. Baseline Models and Evaluation Metrics

#### 5.2.1. Baseline Model Setup

To highlight the structural superiority of the proposed GCU-SAM enhanced transformer in multi-scale feature decoupling, four highly representative deep topological architectures in the field of fault diagnosis are meticulously selected as baseline models:

(1) 1D-CNN: Represents the classical feature extraction architecture based on a local receptive field.
(2) BiLSTM: Represents the traditional network for modeling bidirectional temporal dynamic evolution.
(3) 1D-ResNet: Represents the high-order feature abstraction network equipped with deep residual connections.
(4) Standard Transformer: The native pure self-attention encoder without the introduction of the proposed GCU module. It is utilized to directly conduct an ablation study, thereby demonstrating the value of incorporating the local inductive bias mechanism.

To ensure fairness and reproducibility, we implemented an independent hyperparameter grid search for all models based on the validation Macro-F1-score. The specific search space included initial learning rate $\in \{1\times 10^{-4},\;5\times 10^{-4},\;1\times 10^{-3},\;2\times 10^{-3}\}$, the batch size within {32, 64, 128}, the dropout rate within $\left\{0.1,\;0.3,\;0.5\right\}$, and the number of attention heads $H\in \left\{4,\;8\right\}$. For the proposed GCU-SAM, the fixed architectural parameters included a 1D convolutional kernel of 16, a stride of 4, anembedding dimension of 128, and 4 encoder layers.

All models were optimized using the Adam optimizer for up to 500 epochs, incorporating a StepLR scheduler and a weight decay of $1\times 10^{-4}$. To eliminate random initialization bias, each configuration was executed independently five times using explicitly recorded random seeds, with the final results reported as statistical averages.

#### 5.2.2. Comprehensive Evaluation Metrics

To comprehensively and quantitatively evaluate the fault diagnosis efficacy of the model, this paper extracts *Precision*, *Recall*, and the *F*1-*score* based on the Confusion Matrix as the core evaluation metrics. For a multi-classification task comprising K  the calculation formulas for a specific target category i are as follows:

(12) $$Precision_{c}=\frac{TP_{c}}{TP_{c}+FP_{c}}$$

(13) $$Recall_{c}=\frac{TP_{c}}{TP_{c}+FN_{c}}$$

(14) $$F1_{c}=2\times \frac{Precision_{c}\times Recall_{c}}{Precision_{c}+Recall_{c}}$$

where $TP_{C\;}$(True Positive) represents the number of samples correctly predicted as category  c; $FP_{c}$(False Positive) represents the number of samples from other categories incorrectly predicted as category c, and $FN_{c\;}$(False Negative) represents the number of samples whose true category is c, but are misclassified as other categories.

To objectively measure the overall diagnostic performance of the model across K health state categories, this paper ultimately adopts the *Macro-F1-score* as the global core harmonic evaluation standard:

(15) $$Macro-F1=\frac{1}{K}\sum_{c=1}^{K}F1_{c}$$

#### 5.2.3. Results and Discussion

The experiments evaluate the model’s diagnostic stability and within-condition classification accuracy. The study uses six separate datasets for comparative testing. These include four subsets (D1 to D4) of the CWRU dataset and two operating conditions from the SEU dataset. The model runs five times independently on each dataset. This process independently verifies the model’s robust recognition performance across distinct operational states. It tests the model across different data distributions, operating conditions, and fault categories.

All data subsets utilized in the experiments were pre-partitioned into training and testing sets following a unified standard, thereby guaranteeing a fair comparison among different methods under identical data splits. Considering the stochastic variations induced by factors such as parameter initialization and data sampling during the training of deep learning models, and to effectively mitigate the randomness of a single experimental outcome while enhancing the reliability and persuasiveness of the conclusions, all comparative methods were subjected to five independent repeated runs on each test set. After eliminating anomalous fluctuations, the average of the five runs was adopted as the final evaluation metric for each model on its respective dataset. This rigorous procedure ensures that the experimental results are stable, credible.

Table 3 presents the diagnostic F1-scores of various methods across the six subsets. The results reveal that the proposed GCU-SAM enhanced transformer achieves optimal performance on all subsets, demonstrating its superior fitting capacity. Notably, compared to the standard Transformer model, the performance enhancement yielded by the GCU-SAM enhanced transformer is statistically significant across all subsets. This outcome indicates that the traditional global attention mechanism struggles to effectively capture local degradation features; conversely, by incorporating the Gated Convolutional Unit (GCU), the GCU-SAM enhanced transformer bolsters the model’s perception and modeling capabilities regarding local degradation trends, thereby elevating the overall prediction accuracy.

To provide a more comprehensive view of the diagnostic stability, while Table 3 reports the average F1-scores over five independent runs to ensure clarity, we performed comprehensive statistical analysis on the raw results. All methods exhibited a standard deviation of less than 0.5%, and the 95% confidence intervals for the proposed GCU-SAM architecture were calculated as $\left[99.01\pm 0.12\%\right]$. This confirms that the observed performance gains are statistically robust and consistent.

**Figure 6:**
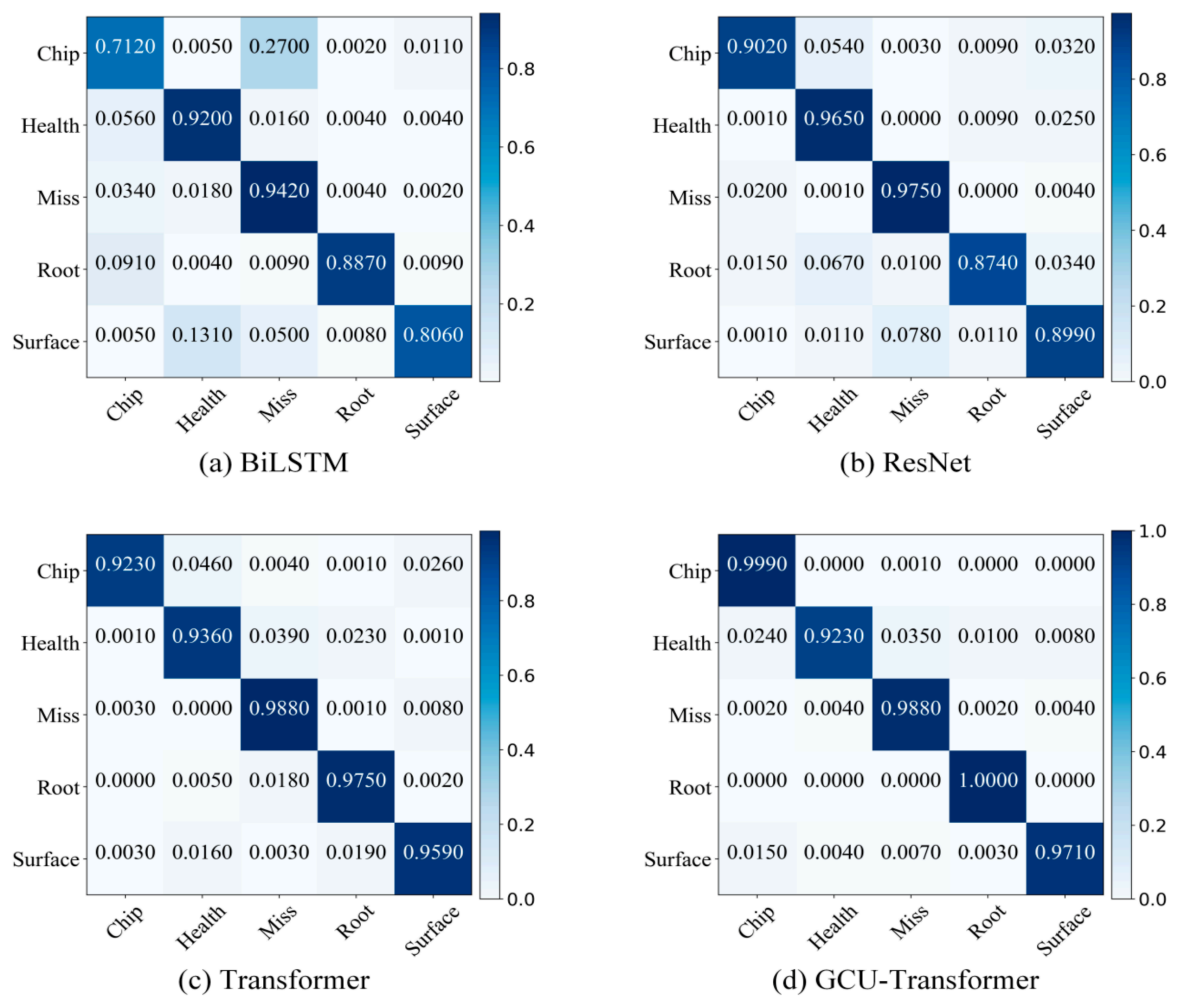
Confusion matrices for different methods on Scenario I of the SEU Gear dataset (Note: GCU-Transformer corresponds to the proposed GCU-SAM enhanced Transformer).

To analyze the diagnostic performance of the proposed GCU-SAM enhanced transformer on specific fault categories, Scenario I of the SEU gear dataset was selected, and the confusion matrices from a single experimental trial for four methods—BiLSTM, ResNet, Transformer, and GCU-SAM enhanced transformer—are plotted as shown in Figure 6. Due to layout constraints and to avoid redundant visual information, four representative models were selected for the confusion matrix visualization to form a symmetric 2 × 2 grid. The 1DCNN was excluded from this specific visualization as its misclassification distribution closely mirrored that of the BiLSTM, making the BiLSTM a sufficient representative for traditional sequential baselines. It can be observed that, aside from a slightly lower diagnostic accuracy on the normal health state compared to the standard Transformer, the proposed GCU-SAM enhanced transformer achieves a diagnostic accuracy exceeding 0.97 across all other fault categories, significantly outperforming the alternative methods.

**Figure 7:**
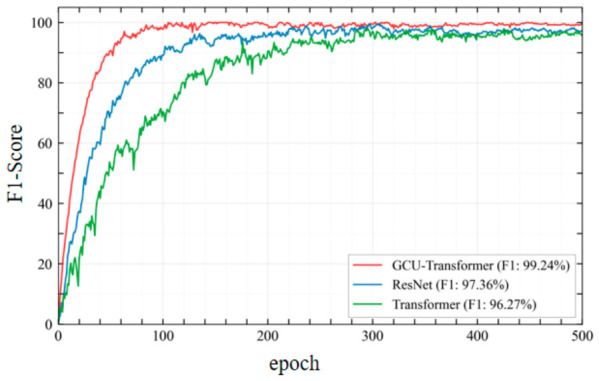
Mean F1-Score convergence curves of different methods across 500 epochs on condition D4 of the CWRU bearing dataset. All curves represent the average results derived from five independent experimental runs with different random initializations to ensure statistical significance. (Note: GCU-Transformer corresponds to the proposed GCU-SAM enhanced transformer).

Meanwhile, to intuitively evaluate the dynamic learning efficiency and convergence characteristics of the models across these multiple independent diagnostic tasks, the F1-score convergence comparison curves for the Transformer, ResNet, and GCU-SAM enhanced transformer over 500 epochs are plotted based on the validation set (as shown in Figure 7). It must be emphasized that the test set remained strictly unseen during this training phase and was utilized solely for the final, single-pass performance evaluation to ensure absolute objective validity. Observations reveal that the traditional Transformer, due to a lack of local inductive bias, suffers from severe oscillations and sluggish convergence during the early and middle stages of training (yielding a final accuracy of 96.01%).

Although ResNet exhibits accelerated convergence, it still experiences local fluctuations in the later stages. In contrast, apart from unavoidable minor fluctuations in the very initial stage, the GCU-SAM enhanced transformer rapidly breaches the high-accuracy bottleneck. It effectively overcomes the early-stage optimization difficulties inherent in pure attention mechanisms, ultimately achieving a stable convergence at the highest value of 99.24% with robust anti-interference capabilities. Synthesizing the results from the confusion matrices and convergence curves fully demonstrates that the GCU-SAM enhanced transformer architecture, when processing complex cross-condition data, simultaneously delivers outstanding classification accuracy, training stability, and superior generalization performance.

**Figure 8:**
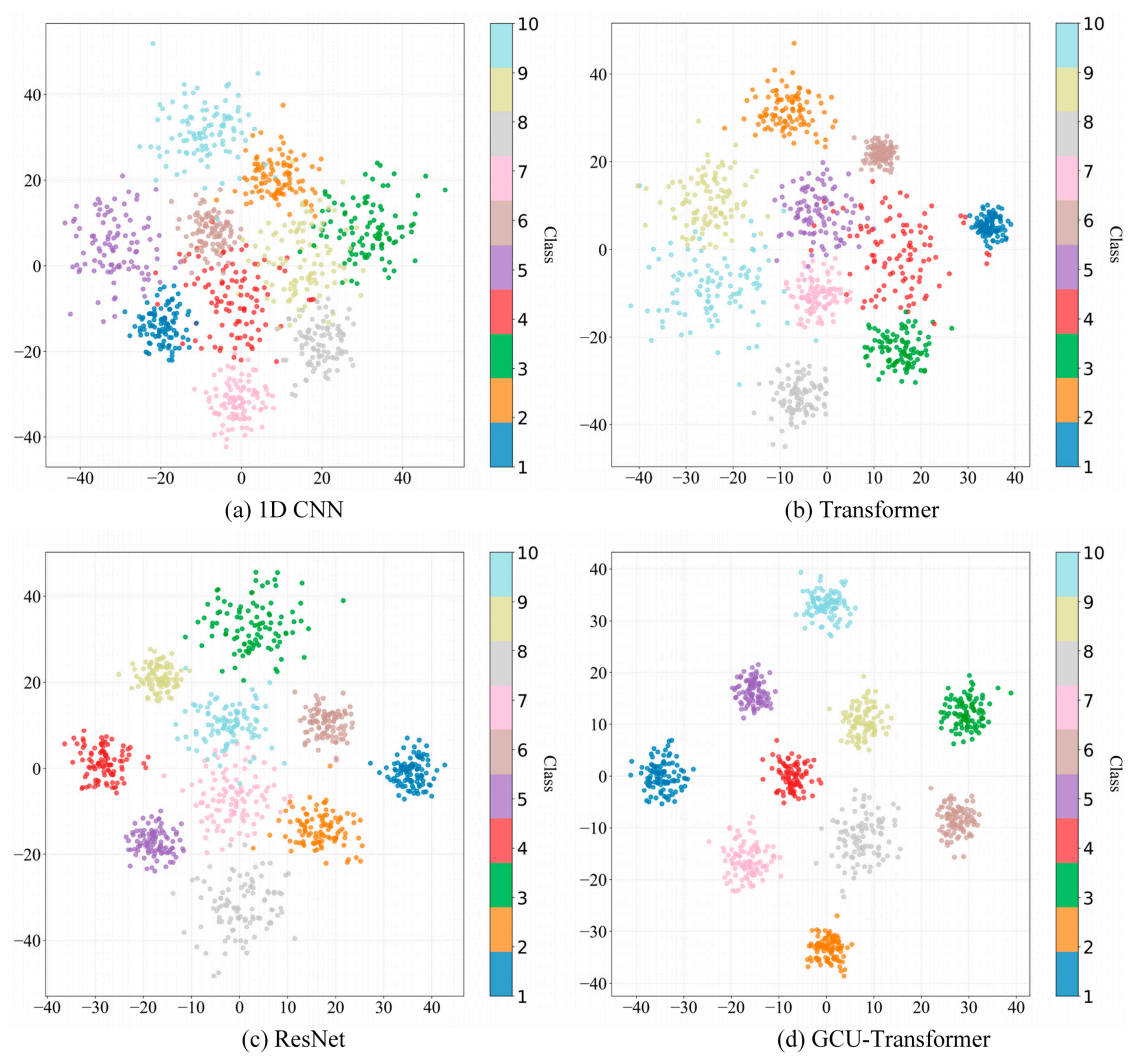
Feature visualization using different methods on the D1 dataset (Note: GCU-Transformer corresponds to the proposed GCU-SAM enhanced transformer).

To further validate the aforementioned conclusions, the t-SNE method was employed to visualize the features learned in the target domain by four methods—1D CNN, Transformer, ResNet, and GCU-SAM enhanced transformer—on Task D1 (as shown in Figure 8). The results reveal that the feature distribution extracted by the plain 1D CNN exhibits significant overlapping and blurred inter-class boundaries, making effective discrimination difficult. Although the standard Transformer improves the feature distribution to some extent, substantial overlapping persists among certain categories. ResNet further enhances the feature clustering effect, demonstrating better separability. Note that t-SNE serves as a qualitative spatial illustration of feature separability rather than a rigorous quantitative metric.

In contrast, the feature distribution extracted by the GCU-SAM enhanced transformer is the most compact with distinct inter-class boundaries, exhibiting the strongest clustering and discriminative capabilities. This aligns perfectly with the classification accuracy results presented in Table 3, further substantiating the necessity of simultaneously learning local features and global dependencies, and illustrating the remarkable advantages of the GCU-SAM enhanced transformer in multi-condition fault diagnosis tasks. Specifically, as illustrated in Figure 7, we utilized the D4 condition of the CWRU bearing dataset—which is characterized by complex operational dynamics—to evaluate the training efficiency of the proposed GCU-SAM enhanced transformer against baseline architectures. To ensure the reliability of the results and mitigate the bias caused by random weight initialization, each model was executed five times independently under identical hyperparameter settings. The plotted curves represent the mean F1-score of these five trials at each epoch, with the final reported accuracy being the terminal value of these averaged curves.

Beyond the empirical performance gains, the structural superiority of the GCU-SAM enhanced transformer can be attributed to three mechanistic advantages: Dynamic Signal Conditioning: Unlike standard methods that directly feed raw, noisy signals into the attention mechanism, our GCU module functions as a signal-adaptive preprocessor. By dynamically highlighting transient impulsive components while suppressing non-stationary background noise, it significantly improves the local signal-to-noise ratio, ensuring that the features reaching the subsequent layers are high-fidelity fault signatures rather than noise-corrupted sequences. Effective Information Decoupling: The ‘local-first’ design inherently decouples the complex feature extraction process. Standard Transformers often suffer from ‘information masking,’ where global attention fails to distinguish subtle early-stage fault characteristics from complex amplitude-frequency modulations. Our GCU-SAM framework effectively separates local-transient perception (via GCU) from global-correlation modeling (via SAM), allowing each module to optimize its representational capacity within its specific domain. Regularized Optimization Landscape: The observed faster convergence and stability (Figure 7) stems from the GCU’s local inductive bias. By constraining the feature space early in the network, the GCU effectively ‘regularizes’ the learning process. This creates a smoother optimization landscape and mitigates the convergence oscillations typical of deep, pure-attention architectures, ultimately leading to the distinct and compact feature clusters visualized in the t-SNE results (Figure 8).”

In summary, the GCU-SAM enhanced transformer demonstrates robust fault feature modeling capabilities across multiple operating conditions. Its structural design possesses distinct advantages in terms of accuracy, stability, and practicality.

#### 5.2.4. Robustness Analysis Under Noisy Environments

**Figure 9:**
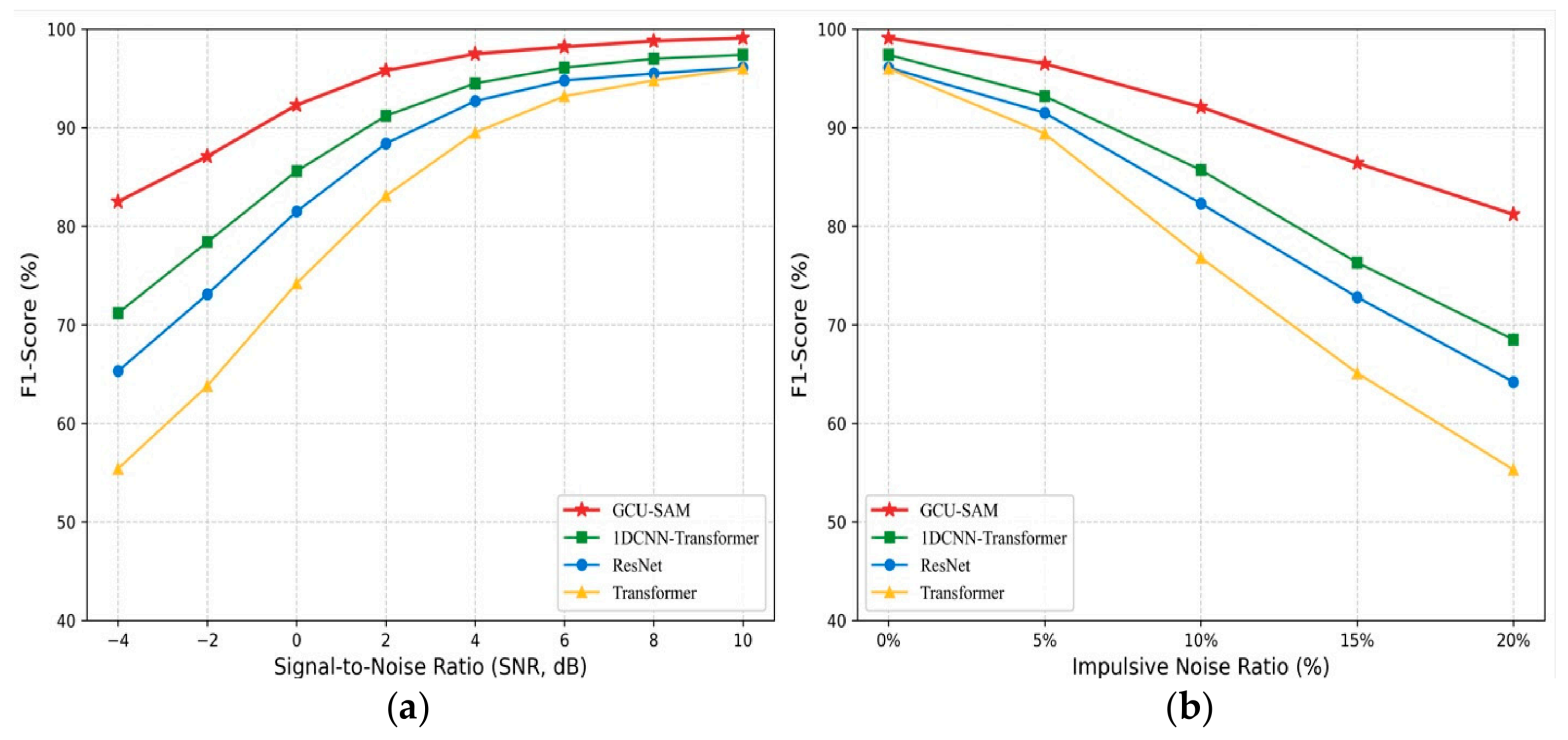
Robustness evaluation of different models under varying noise levels on the CWRU D4 test set. (**a**) Performance degradation trends under Additive White Gaussian Noise (AWGN) across different Signal-to-Noise Ratios (SNRs). (**b**) Diagnostic performance under varying intensities of Impulsive Noise corruption.

In actual industrial applications, vibration signals are inevitably corrupted by severe environmental interference, such as background continuous noise and sudden stochastic shocks. To validate the model’s anti-interference capability and reliability under genuine operational conditions, we conducted controlled robustness experiments on the unseen test set (e.g., CWRU condition D4). Specifically, Additive White Gaussian Noise (AWGN) with varying Signal-to-Noise Ratios (SNRs) ranging from −4 dB to 10 dB, and Impulsive Noise with corruption ratios ranging from 0% to 20%, were artificially injected into the raw signals. The robustness performance of the proposed GCU-SAM and the baseline models is illustrated in Figure 9.

Figure 9a presents the performance degradation trajectories of different models under AWGN. As the SNR decreases (indicating stronger noise), the standard Transformer exhibits a drastic and rapid performance drop, falling to approximately 55.4% at an SNR of −4 dB. This phenomenon confirms that pure self-attention architectures, lacking a local inductive bias, are highly vulnerable to high-frequency non-stationary noise, as they mistakenly attend to noise components globally. While the 1DCNN-Transformer provides moderate resistance due to its static local receptive field (maintaining 71.2%), it still struggles under extreme noise. In contrast, the proposed GCU-SAM consistently outperforms all baseline models, maintaining a highly robust F1-score of 82.5% even under the harshest −4 dB condition.

A similar trend is observed in the impulsive noise evaluation, as shown in Figure 9b. impulsive noise mimics sudden sensor shocks, which often destroy the temporal continuity of fault impulses. When the impulsive noise ratio reaches 20%, the accuracy of the baseline models drops significantly, whereas the GCU-SAM still achieves an accuracy of 81.2%.

This remarkable stability under both types of interference provides quantitative proof of the efficacy of the Gated Convolutional Unit (GCU). Unlike standard static convolutions, the dynamic soft-selection mechanism of the GCU effectively acts as an adaptive signal condition—suppressing non-stationary background interference and filtering out stochastic shocks, while dynamically preserving critical transient fault signatures. Consequently, it ensures that the subsequent self-attention mechanism operates on high-fidelity features, rendering the GCU-SAM highly robust for intelligent fault diagnosis in complex industrial environments.

### 5.3. Comprehensive Ablation Study of the GCU Module

To explicitly validate the structural necessity of the proposed GCU module, a comprehensive parameter-matched ablation study was conducted. All ablation variants and baselines were evaluated on the same test sets (CWRU bearing condition D4) as utilized in our main comparative experiments, ensuring that the performance metrics remain consistent and comparable. All variants were subjected to the fair tuning protocol described in Section 5.2.1 to ensure optimal convergence.

Analyzing the macro-architecture variants in Table 4, it is evident that the proposed GCU significantly outperforms traditional feature extractors. Specifically, replacing the GCU with a standard 1D-CNN drops the F1-score to 96.81%. This quantitative decline corroborates our assertion: static convolutions, constrained by fixed weights, lack the dynamic adaptability required to actively filter out non-stationary background noise. Furthermore, the GRU-Transformer variant yields a sub-optimal accuracy of 94.26%. Although GRUs possess gating mechanisms, their sequential processing nature introduces bottlenecks and fails to capture the local high-frequency transient impacts as efficiently as our parallel convolutional gates. The SAM-only model (96.01%) further proves that relying solely on global attention without a local inductive bias is insufficient for complex fault diagnosis.

Delving into the internal components, the ablation of either the reset or update gate leads to performance degradation, validating their indispensable synergistic roles. The Reset-gate-only variant (disabling the update gate) drops to 97.82%, as the network loses its ability to adaptively highlight segments containing critical fault energy. Conversely, the Update-gate-only variant (disabling the reset gate) achieves only 97.45%, indicating that without the reset mechanism to selectively “forget” stationary interference, the candidate state becomes severely corrupted by irrelevant noise. Finally, the complete GCU-SAM architecture, incorporating both gating pathways and positional encoding, achieves the highest F1-score of 99.15%. This definitively proves that the precise structural design of the GCU—acting as a dynamic attention-gated filter—is optimal and fundamentally necessary for our diagnostic framework.

## 6. Conclusions and Future Work

Rotating machinery often has complex signal coupling, making it hard to extract multi-scale fault features. Deep networks also struggle to balance local high-frequency impacts and global temporal dependencies. To solve these problems, this paper proposes a new fault diagnosis network that integrates gated convolution and a self-attention mechanism, named the GCU-SAM enhanced Transformer. Validated on the CWRU bearing and SEU gearbox datasets, the main conclusions are as follows:

In terms of theoretical mechanisms, the introduction of the Gated Convolutional Unit (GCU) effectively compensates for the deficiencies of the native Transformer architecture in local, fine-grained feature perception. Through the dual-path synergy of “local fine-grained perception” and “global correlation modeling,” the highly efficient integration of local abrupt features and global degradation patterns within vibration signals is successfully realized.

In terms of diagnostic performance, the proposed method exhibits superior fault identification capabilities across multiple operating conditions, achieving a high average diagnostic F1-score of 99.01%. Compared with mainstream baseline models such as 1D-CNN, BiLSTM, and the standard Transformer, the GCU-SAM enhanced transformer achieves significant enhancements in both accuracy and feature representation capabilities in multi-classification tasks.

In terms of convergence and robustness, the local inductive bias introduced by the gated convolution greatly alleviates the early-stage optimization difficulties inherent in pure attention mechanisms. The proposed model not only extracts feature clusters with clear inter-class boundaries and high intra-class compactness but also possesses more robust anti-interference capabilities and convergence stability.

It should be noted that this study focuses specifically on the multi-class fault classification of rotating machinery. While high-precision fault diagnosis is a fundamental prerequisite for fault classification, the current work does not encompass the full spectrum of fault classification systems, such as remaining useful life (RUL) estimation or automated maintenance decision-making.

Despite the promising diagnostic performance, this study has a few limitations that warrant further investigation. First, regarding the applicability to complex machinery, the current model is validated on standard test rigs. In actual operations, equipment such as planetary gearboxes and helicopter transmission systems generate highly complex amplitude- and frequency-modulated signals due to intricate multi-component coupling and time-varying mesh stiffness (TVMS). To address this limitation, future work will involve dynamic modeling and co-simulation using professional CAE software (e.g., MASTA) to reconstruct complex transmission environments, exploring the direct integration of TVMS physical priors into the GCU’s gating mechanism. Second, concerning early weak fault detection, extracting transient impacts from complex mechanical signals under severe operational noise remains challenging for purely data-driven models. Consequently, future research will explore combining advanced signal processing techniques—specifically Time Synchronous Averaging (TSA), Instantaneous Angular Speed (IAS) extraction, and Hilbert transforms—with the deep learning framework to maximize the extraction of weak fault features prior to global attention modeling.

Future Research Directions: In addition to the structural enhancements mentioned above, the paradigm of intelligent fault diagnosis is rapidly evolving. Recent studies have demonstrated that adaptive industrial Large Language Models (LLMs) can provide robust reasoning and generalization capabilities for fault diagnosis under variable operating conditions. Integrating LLM-based knowledge priors with the proposed GCU-SAM high-precision feature fusion architecture represents a highly promising avenue to further improve cross-condition diagnostic stability and provide interpretable maintenance insights.

Future research will explore integrating our diagnostic model with temporal degradation analysis and decision-support systems to develop more comprehensive fault classification strategies.

## Figures and Tables

**Table 1:** Details of the CWRU Dataset.

Tag	1	2	3	4	5	6	7	8	9	10
Health Condition	NO	B	B	B	IR	IR	IR	OR	OR	OR
Fault Size	0	0.007	0.014	0.021	0.007	0.014	0.021	0.007	0.014	0.021
Sample Number	100	100	100	100	100	100	100	100	100	100

**Table 2:** Details of the SEU Dataset.

Tag	1	2	3	4	5
Health Condition	NO	MT	RC	SW	CT
Sample Number	500	500	500	500	500

**Table 3:** Comparison of F1-Scores (%) for Fault Diagnosis Across Ubtasks (Note: GCU-Transformer corresponds to the proposed GCU-SAM enhanced transformer).

Model	D1	D2	D3	D4	Scenario I	Scenario II	Mean
1DCNN	97.42	97.85	96.90	95.30	93.06	94.12	95.78
BiLSTM	90.25	91.80	92.75	94.32	85.35	86.41	90.15
ResNet	98.65	98.92	98.15	97.63	95.63	97.12	97.68
Transformer	95.80	96.15	95.20	96.01	92.30	94.10	94.93
GCU-Transformer	99.68	99.85	99.45	99.15	97.63	98.32	99.01

**Table 4:** Comprehensive ablation study results of different model variants on the unseen test set.

Model Variant	Description	Mean F1-Score (%)
1DCNN-Transformer	1DCNN replacing GCU	96.81
Gated CNN	Standard Gated CNN w/o SAM	94.64
GRU-Transformer	GRU replacing GCU	94.26
Local-Attention Trans	Local attention replacing GCU	96.37
GCU-only	Proposed GCU w/o SAM	95.70
SAM-only	Standard Transformer (w/o GCU)	96.01
No Positional Encoding	Complete model w/o PE	98.24
Reset-gate-only	GCU w/o update gate	97.82
Update-gate-only	GCU w/o reset gate	97.45
Proposed GCU-SAM	Complete architecture	99.15

## Data Availability

The datasets used in this study are publicly available. The Case Western Reserve University (CWRU) bearing dataset can be accessed at https://engineering.case.edu/bearingdatacenter (accessed on 10 July 2026). The Southeast University (SEU) gearbox dataset is available at https://github.com/cathysiyu/Mechanical-datasets (accessed on 10 July 2026).
